# Translation and validation of the Greek versions of the Coronavirus Anxiety Scale, the Obsession with COVID-19 Scale and the Coronavirus Reassurance-Seeking Behaviors Scale

**DOI:** 10.1192/j.eurpsy.2024.554

**Published:** 2024-08-27

**Authors:** M. Bakola, K. S. Kitsou, V. Angelidou, A. Giannakopoulos, M. Drakopoulou, K. Mavridou, P. Kalianezos, K. Argyropoulos, C. Petropoulos, G. Charalambous, P. Gourzis, S. A. Lee, E. Jelastopulu

**Affiliations:** ^1^Department of Public Health, School of Medicine, University of Patras, Patras, Greece; ^2^Postgraduate Program of Health Management, Frederick University of Nicosia, Nicosia, Cyprus; ^3^Department of Mathematics; ^4^Department of Psychiatry, University of Patras, Patras, Greece; ^5^Department of Psychology, Christopher Newport University, Newport News, United States

## Abstract

**Introduction:**

Understanding coronophobia, or the heightened fear and anxiety related to the COVID-19 pandemic, involves assessing physiological, cognitive, and behavioral measures.

**Objectives:**

We aimed to develop a Greek version of the Coronavirus Anxiety Scale (CAS), the Obsession with COVID-19 Scale (OCS), and the Coronavirus Reassurance-Seeking Behaviors Scale (CRBS), to identify groups that appear vulnerable to this form of pandemic-related anxiety.

**Methods:**

We conducted a cross-sectional online study from February to April 2021 in Greek-speaking people living in Cyprus. Participants completed sociodemographic questions and questions related to COVID-19, the CAS, OCS, and the CRBS. All three scales are rated on a 5-point scale, from 0 (not at all) to 4 (nearly every day). For CAS, a score ≥ 9 indicates probable dysfunctional coronavirus-related anxiety, for OCS a ≥ 7 score indicates probable dysfunctional thinking about COVID-19, and for CRBS score ≥ 12 suggests above-average reassurance-seeking activity.

**Results:**

A total of 405 adults (66.4% women) from Cyprus participated in this study. The results of this study demonstrate that these Greek adapted measures have adequate reliability (Cronbach’s alphas >0.70) and factor structure (exploratory and confirmatory factor analysis support). However, only the CAS demonstrated both convergent and divergent validity. Education personnel, housekeepers, and older adults were also found to have higher coronavirus anxiety relative to their counterparts.

**Conclusions:**

The findings of this research support the use of these coronaphobia scales in Cyprus and other Greek-speaking populations. Assessing the potential for fear-driven behaviors may be of great benefit to both clinicians and researchers, helping to identify individuals at risk, adapt interventions, and improve our understanding of the psychological consequences of surviving a public health emergency.

**Disclosure of Interest:**

None Declared

